# The effect of in vitro simulated colonic pH gradients on microbial activity and metabolite production using common prebiotics as substrates

**DOI:** 10.1186/s12866-024-03235-2

**Published:** 2024-03-11

**Authors:** Zhuqing Xie, Weiwei He, Alex Gobbi, Hanne Christine Bertram, Dennis Sandris Nielsen

**Affiliations:** 1 Department of Food Science, University of Copenhagen, Frederiksberg, Denmark; 2 Department of Food Science, Aarhus University, Aarhus N, Denmark; 3 Present Address: State Key Laboratory of Food Science and Technology, Nanchang University, Nanchang, China; 4 Department of Plant and Environmental Sciences, University of Copenhagen, Frederiksberg, Denmark; 5 Present Address: European Food and Safety Authority, Parma, Italy

**Keywords:** Colonic pH, In vitro colonic fermentation, Gut microbiota, Short-chain fatty acids, Prebiotics

## Abstract

**Background:**

The interplay between gut microbiota (GM) and the metabolization of dietary components leading to the production of short-chain fatty acids (SCFAs) is affected by a range of factors including colonic pH and carbohydrate source. However, there is still only limited knowledge on how the GM activity and metabolite production in the gastrointestinal tract could be influenced by pH and the pH gradient increases along the colon.

**Results:**

Here we investigate the effect of pH gradients corresponding to levels typically found in the colon on GM composition and metabolite production using substrates inulin, lactose, galactooligosaccharides (GOS), and fructooligosaccharide (FOS) in an in vitro colon setup. We investigated 3 different pH regimes (low, 5.2 increasing to 6.4; medium, 5.6 increasing to 6.8 and high, 6.0 increasing to 7.2) for each fecal inoculum and found that colonic pH gradients significantly influenced in vitro simulated GM structure, but the influence of fecal donor and substrate was more pronounced. Low pH regimes strongly influenced GM with the decreased relative abundance of *Bacteroides* spp. and increased *Bifidobacterium* spp. Higher in vitro simulated colonic pH promoted the production of SCFAs in a donor- and substrate-dependent manner. The butyrate producer *Butyricimonas* was enriched at higher pH conditions, where also butyrate production was increased for inulin. The relative abundance of *Phascolarctobacterium*, *Bacteroides*, and *Rikenellaceae* also increased at higher colonic pH, which was accompanied by increased production of propionate with GOS and FOS as substrates.

**Conclusions:**

Together, our results show that colonic substrates such as dietary fibres influence GM composition and metabolite production, not only by being selectively utilized by specific microbes, but also because of their SCFA production, which in turn also influences colonic pH and overall GM composition and activity. Our work provides details about the effect of the gradients of rising pH from the proximal to distal colon on fermenting dietary substrates in vitro and highlights the importance of considering pH in GM research.

**Supplementary Information:**

The online version contains supplementary material available at 10.1186/s12866-024-03235-2.

## Background

The human colon harbors a complex microbial community, the gut microbiota (GM), that influences host physiology and metabolism. The GM aids in utilizing otherwise indigestible dietary fibers through fermentation thereby generating a variety of metabolites including short-chain fatty acids (SCFAs), succinate, lactate, methane, and hydrogen [1]. Acetate, propionate, and butyrate are the major SCFAs and are of particular interest not only as an energy source, but also via exhibiting benefits to the host such as enhancing satiety, suppressing appetite, and modulating inflammation [2]. Colonic pH differs from person to person as well as between different colonic segments, where the pH of the proximal (5.4–5.9) and transverse (6.1–6.4) colon is lower than the distal colon which is close to neutrality (6.4–8.0) [3–5]. The abundance of indigestible carbohydrates in the proximal colon favor saccharolytic fermentation leading to SCFA production which decreases the colonic pH [6]. In contrast, proteolytic fermentation dominates the distal colon where the available carbohydrates are depleted leading to an increase in pH [7]. This might influence host health and physiology, where e.g. cations (mainly Ca^2+^) have higher bioavailability at lower pH [8] and with low colonic pH generally protecting against microbial pathogens [9].

The interplay between GM and the host via the degradation of fibers to produce SCFAs is highly complicated and can be affected by several factors, including colonic pH and carbohydrate source [10, 11]. Changes in pH influence not only the bacterial community [12] but also metabolite production [11], which further connects with colonic function [13, 14]. By determining the fecal concentration of SCFA across several human studies, LaBouyer et al. (2022) [15] found that the fraction of total SCFA constituted by butyrate increased with absolute SCFA concentration which in turn correlated with lower pH. This could indicate that butyrate production is favored by lowered pH, but it could vice versa also indicate that when total SCFA concentration is high, pH is obviously relatively low. Acetate is also involved in gut microbial cross-feeding networks, where it can serve as a substrate for many butyrate producers [15, 16]. It has been found that acidic pH overall supports the growth of bacteria belonging to *Firmicutes* phylum whereas many *Bacteroides* members are more sensitive to acidity [17]. *Escherichia coli*, a species that constitute both commensal and pathogenic members in the human colon, can be inhibited by acidic pH (5.5) under in vitro simulated colonic environments, whereas bifidobacteria and lactobacilli are favored by more acidic conditions [17, 18]. Furthermore, colonic pH has been reported to influence the production of specific metabolites with butyrogenic reactions being favored at a slightly acidic pH, which is in contrast with propionate production that in general is increased at neutral pHs [19]. Acetate on the other hand can readily be produced at different pHs via the activity of various acetate-producing microbial species having different pH optima [10, 20]. As a precursor for the production of other SCFAs, especially propionate and butyrate, lactate usually does not accumulate in the colon to any larger extent, and its concentration is closely related to the abundance of lactate-utilizing bacteria [21]. However, some of the lactate utilizers are rather sensitive to acidic pH which may result in lactate accumulation under moderately acidic environments [22].

The carbohydrate source is another factor that influences metabolite production and GM response. Reichardt et al. [19] performed in vitro simulated colon batch fermentations of 15 different dietary fibers including glucans, pectins, hemicellulose, and fructans at two starting pH values (5.5 and 6.5), and found that butyrate made up a larger fraction of the produced SCFAs at the lower pH (5.5) for most substrates, whereas propionate production in general was impaired at the lower pH. Chung et al. [11] investigated SCFA production and microbial community response to inulin or pectin as substrate in pH-controlled fermentors inoculated with fecal matter and found that reducing pH from 6.9 to 5.5 promoted the abundance of *Faecalibacterium prausnitzii* replacing otherwise dominating *Bacteroides* spp. Ilhan et al. [23] used glucose, fructose, or cellobiose as the single carbon sources in batch fermentations using fecal slurry as inoculum under three different starting pHs (6.0, 6.5, and 6.9). It was found that pH had substantial impact on lactate utilizers and producers, resulting in pronounced lactate accumulation at pH 6.0. Besides, microbial diversity was driven not only by pH but also by carbon source, as fructose and cellobiose have higher microbial richness at higher starting pH which was not observed with glucose cultures. These findings suggest that in addition to substrate type, gut environment, especially pH, can also influence the metabolites being produced and the interspecies interactions in the gut.

Inulin and fructooligosaccharides (FOS) are typical prebiotics where the fructose units are joined by β(1,2)-linkages. Interestingly, the fermentation patterns of inulin with a degree of polymerization (DP) less than 10 has been found to be relatively close to FOS [24, 25]. Galactooligosaccharides (GOS) are another type of prebiotics, where the DP varies quite a lot with being composed of 2–9 galactose units and a terminal glucose. All 3 mentioned compounds are considered as prebiotics, promoting especially *Bifidobacterium* spp. but also lactobacilli and other putatively beneficial GM members [26]. As the carbohydrate source in milk, lactose is composed of glucose and galactose bound by a β(1,4)-glycosidic linkage. While still controversial, lactose has been proposed to act as a prebiotic for lactose mal-absorbers [27, 28]. It is well-described that substrate and colonic pH influence GM activity and metabolite production, but it is still only partly understood how the composition and activity of the colonic microbial community is influenced by colonic pH and the pH gradient increases along the colon, and how this in turn will influence SCFA production. To address this, we simulated in vitro the colonic fermentation of common prebiotics (inulin, FOS, GOS as well as the easier degradable lactose) as influenced by physiologically relevant gradients of rising pH during 24 h of fermentation and investigated the changes in microbial composition and SCFA production between these substrates. Fresh stool samples from three donors with different fecal pH values (6.4, 6.8, and 7.2, respectively) were collected, and three dynamic pH programs (proximal to the distal colon) for each inoculum during 24 h of fermentation were designed which represented low (5.2–6.4), medium (5.6–6.8) and high (6.0–7.2) colonic pH gradients. Metabolite outcomes after 24 h of fermentation for the individual fecal slurries under the three different colonic pH regimes were examined by ^1^H NMR metabolomics, and GM shifts were traced using 16S rRNA gene amplicon sequencing. We hypothesized that colon pH gradients influence microbial activity and hence also microbiome composition and SCFA production. Together, our work provides details about the effect of the gradients of rising pH from the proximal to distal colon on fermenting dietary substrates in vitro and highlights the importance of considering colonic pH in GM research.

## Results

### Colonic pH gradients significantly influenced in vitro simulated GM structure, but the influence of fecal donor and substrate was more pronounced

**Fig. 1:**
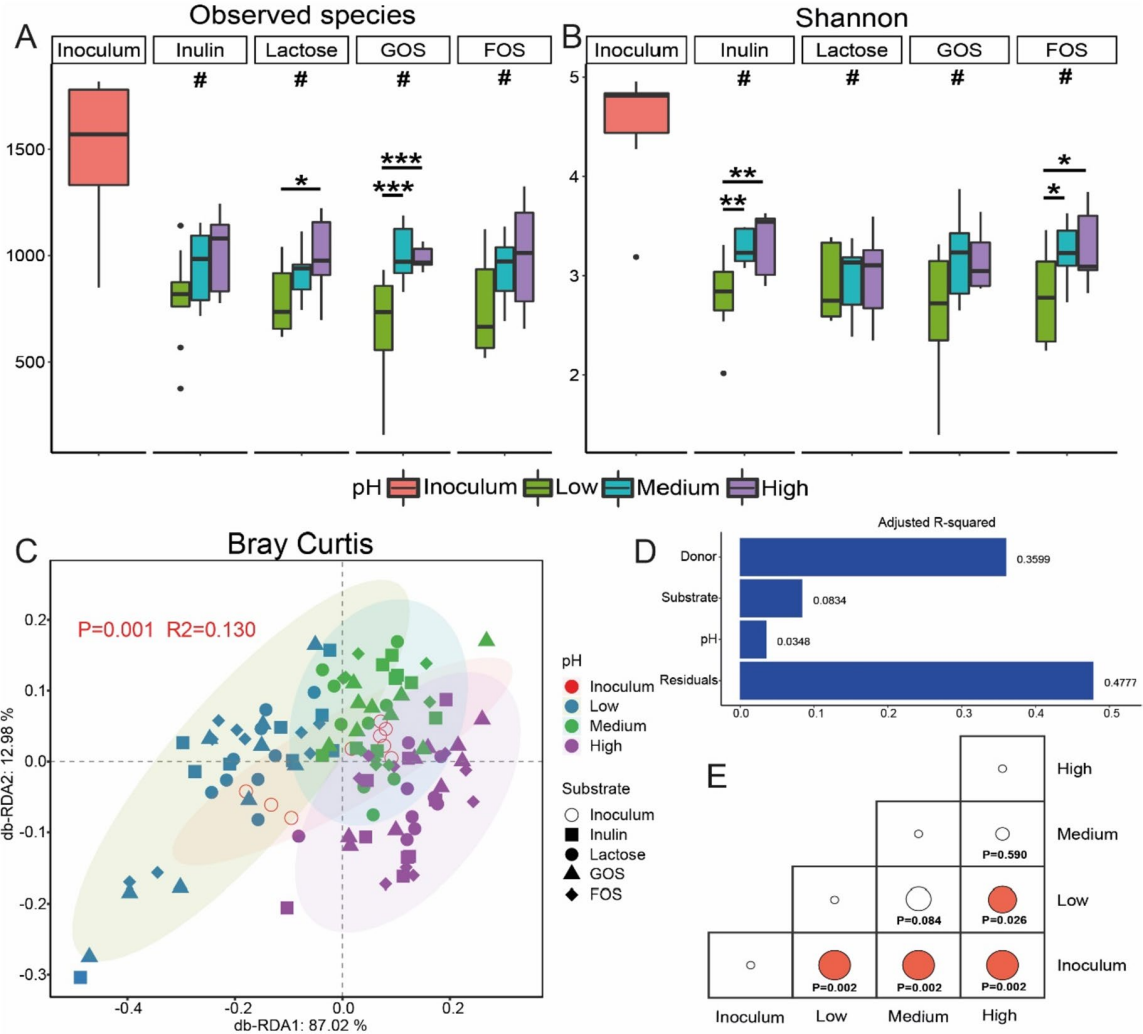
The influence of colonic pH on GM diversity after 24 h of fermentation. Observed species (**A**) and Shannon index (**B**) of the microbial community, Db-RDA biplot (**C**) showing microbial variance explained by colonic pH with adjusted R2 (**D**) and pairwise PERMANOVA tests (**E**) on Bray Curtis metrics. A t-test was applied to determine the influence of pH on alpha diversity for three inocula with a single substrate. Benjamin-Hochberg FDR (false discovery rate) correction was adopted for multiple testing. Significant differences between changed colonic pH are labeled with * (*p* < 0.05), ** (*p* < 0.01) and *** (*p* < 0.001), respectively. Significant differences relative to inoculum are labeled with # (*p* < 0.05)

**Fig. 2:**
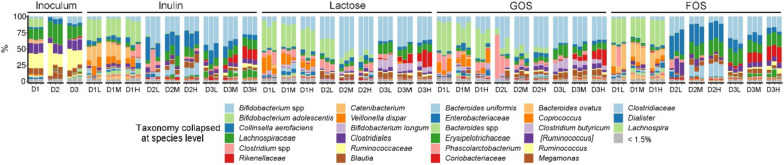
Summarized species-level gut microbiota composition of individual fermentation samples with different substrates. D, donor; L, low pH from 5.2-6.4; M, medium pH from 5.6-6.8; H, high pH from 6.0-7.2

The microbiome composition of the in vitro simulated colonic fermentations were determined by 16S rRNA gene amplicon sequencing. The number of sequencing reads was rarefied to 100,000 reads pr. sample (see Fig. S1 for rarefaction curves). A total of 4,212 zero radius Operational Taxonomic Units (zOTUs) were obtained and summarized at species level. After 24 h of in vitro batch fermentation, the number of observed species as well as the Shannon diversity index were higher for the higher colonic pH gradients (see Fig. 1 for average across donors and Fig. S2 for individual donors). Bray–Curtis dissimilarity-based db-RDA analysis (Fig. 1C) showed clear groupings of the in vitro simulated colonic fermentation samples according to pH, which was confirmed by pairwise PERMANOVA (Fig. 1E and Fig. S3C-D). However, pH explained only 3.48% of the total variance (Fig. 1D) relative to the effect of the donor (35.99%) and substrate (8.34%). In accordance with this, PCoA plots based on both Bray–Curtis dissimilarity and Jaccard similarity metrics showed strong donor effects (Fig. S3 A-B) which is also reflected by the actual microbiome composition as seen in Fig. 2. Overall, all four tested substrates led to a higher relative abundance of bifidobacteria relative to inoculums. Low in vitro colonic pH gradient especially promoted the proliferation of bifidobacteria when inulin was used as substrate (Fig. S4).

### Low in vitro simulated colonic pH strongly influenced microbiome composition

**Fig. 3:**
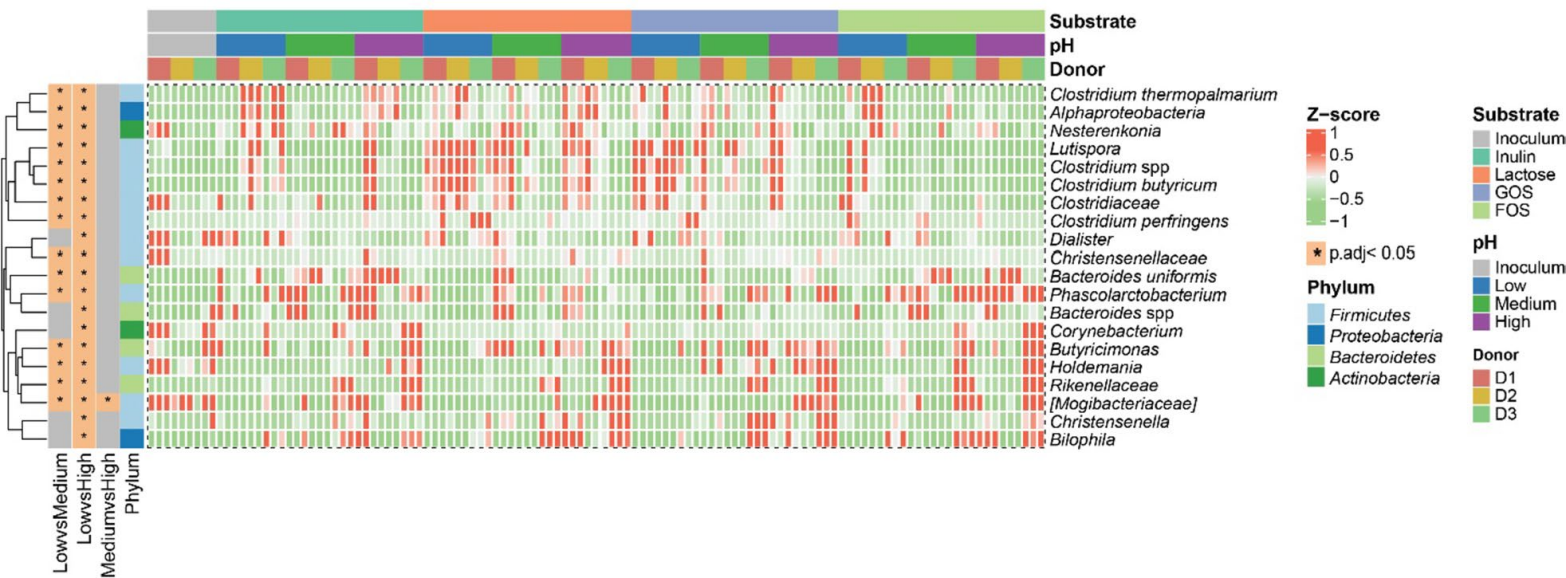
Differentially abundant taxa between changed colonic pH. The top 20 differentially abundant taxa with the lowest p-adjust value were selected by Deseq2 and labeled with * in the heatmap, indicating the significantly altered taxa from the respective between-group comparison. Row-wise Z-score scaling was conducted in the heatmap visualization, showing the normalized relative abundance by the mean of the specific taxa across all samples. D, donor

Differential abundance analysis (Deseq2) was carried out to determine compositional differences in the in vitro simulated GMs under different colonic pH regimes (Fig. 3). When comparing the effect of pH, it is evident that low pH (5.2 increasing to 6.4) strongly influences the microbiome, while only a *Mogibacteriaceae* member was found to differ between the medium (5.6 increasing to 6.8) and the high pH (6.0 increasing to 7.2) regime. In summary, acidic pH promoted most *Firmicutes* members (*Clostridium* members, *Lutispora*, and *Dialister*) but suppressed the growth of phylum *Bacteroidetes* members including *Bacteroides*, *Butyricimonas*, and *Rikenellaceae* relative to high pH conditions. Low pH conditions led to the decreased relative abundance of *Christensenellaceae*, *Phascolarctobacterium, Holdemania*, *Mogibacteriaceae*, and *Christensenella* for all four tested substrates relative to the medium and high pH regimes.

### Higher in vitro simulated colonic pH promoted the production of SCFAs in a donor- and substrate-dependent manner

**Fig. 4:**
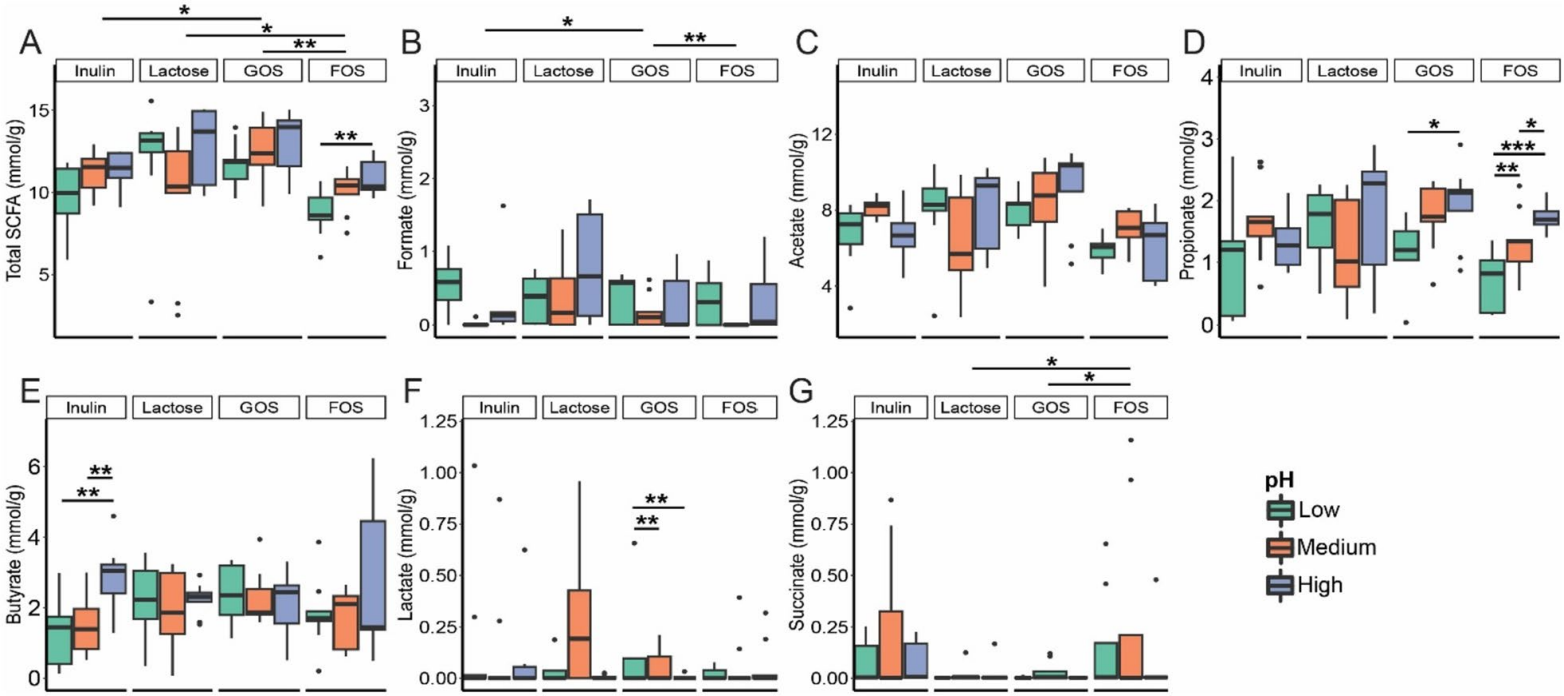
The influence of colonic pH on metabolite production (in mmol per gram of substrate) after 24 h of fermentation. **A**-**F** represents total SCFA, acetate, propionate, butyrate, formate, and lactate production, respectively. A t-test was applied to determine the influence of pH on metabolite production with a single substrate or differences in metabolite production between substrates. Benjamin-Hochberg FDR (false discovery rate) correction was adopted for multiple testing. Significant differences between changed colonic pH are labelled with * (*p* < 0.05), ** (*p* < 0.01) and *** (*p* < 0.001), respectively

Higher in vitro simulated colonic pH favored SCFA production for all substrates tested, and higher fermentation pH of FOS liberated significantly more SCFA than low or medium pH (*p* < 0.05) (Fig. 4). At higher simulated pH, inulin fermentation promoted increased butyrate formation (*p* > 0.01), and FOS (*p* < 0.001) and GOS (*p* < 0.05) increased propionate formation (Fig. 4), which was confirmed by the OPLS-DA model and S-line plots of NMR spectra (Table S1 and Fig. S5). Acetate production appeared to be less influenced by pH, as no significant differences in acetate concentrations were observed between the different pH regimes. Formate, lactate and succinate, three intermediate products/substrates in SCFAs formation, were only detected in relatively low concentrations. Interestingly, lactate accumulation during GOS-based fermentations was reversed by increased colonic pH (Fig. 4F). Again, donor-specific differences were observed with respect to both total SCFA formation as well as specific SCFAs like acetate and propionate being promoted by higher pH with GOS as the substrate for donors 2 and 3, whereas SCFA production by donor 1 was not affected by pH to any larger extent when grown on GOS (Fig. S6). Besides, lactose presented apparent individualized SCFA production, where the corresponding pH regime for Donor 1 (low fecal pH) and Donor 3 (high fecal pH) favored higher SCFA (acetate) production. These observations at least in part likely also reflect that individuals with high or low colonic pH also harbor gut microbial communities adapted to these conditions.

### Propionate and butyrate production is associated with specific bacterial taxa and pH-gradients

**Fig. 5:**
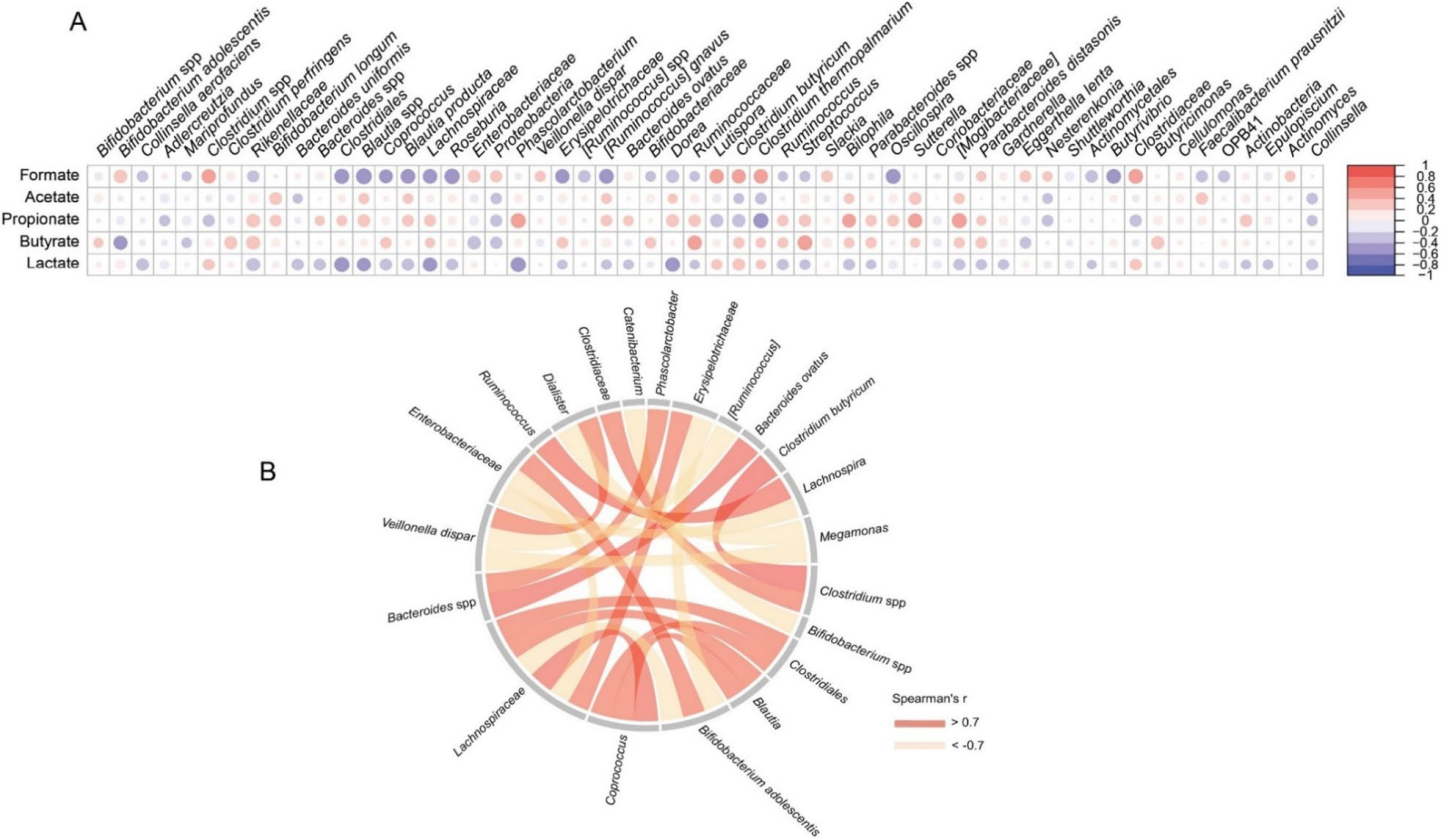
Correlation between microbial taxa relative abundance and metabolites (formate, acetate, propionate, butyrate, and lactate) concentrations (**A**) and inter-species co-occurrence (**B**). The minimal prevalence (among the sequenced samples) of one given microbial taxa was set to 30%. Species with a mean relative abundance >1.5% were chosen for co-occurrence analysis, and Spearman's rank correlation coefficient of > 0.7 is shown in the chord diagram

Associations between specific bacterial species and metabolite production were determined by Pearson’s correlation analysis and 138 significant pairs were found (Table S2). As seen from Fig. 5A, the high relative abundance of *Mogibacteriaceae*, *Phascolarctobacterium*, *Rikenellaceae*, and *Bacteroides* spp. at the high in vitro simulated colonic pH was positively correlated with propionate production, while *Butyricimonas*, which also showed higher relative abundance with higher pH, presented a positive relationship with butyrate concentration. Co-occurrence analysis (Fig. 5B) was conducted to explore the interactions among species where the relative abundance varied with simulated colonic pH. *Phascolarctobacter* had a strong positive correlation with *Bacteroides* spp, and *Lachnosira* was positively correlated with *Ruminococcus*. Besides, *Lachnospiraceae* was positively correlated with *Clostridiales* and *Blautia* but inversely correlated with *Bifidobacterium adolescentis*. *Coprococcus* was positively correlated with *Lachnospiraceae*, *Clostridiales*, and *Blautia*, respectively. In summary, several abundant microbial members (relative abundance > 1.5%) with strong positive co-occurrence (e.g. *Lachnospiraceae*, *Ruminococcus*, *Clostridiales*, *Coprococcus*, and *Blautia*) consistently correlated with more propionate/butyrate production (Fig. 5A).

## Discussion

Indigestible dietary compounds such as fibers and other complex carbohydrates benefit host physiology via the colonic production of key bacterial metabolites like SCFAs [29], which in turn also influence colonic pH [30]. Wide inter-individual differences in colonic pH (5.3–8.0) and gut transit time (0.1–80 h) have been reported [5], but the influence of factors such as the dynamic pH increase from the proximal to distal colon on interspecies bacterial balance and metabolites formation remains poorly understood. To investigate this, we employed an in vitro colonic model with different pH gradients for individual inoculum simulating the dynamic pH conditions of the ascending, transverse, and descending colon when fermenting substrates. Fresh stool samples from three healthy adults with different fecal pH-levels (6.4, 6.8, and 7.2) were used, which represent individuals with low, intermediate, and high colonic pH, respectively.

The alpha diversity indexes observed species and Shannon Diversity were higher at a higher colonic pH, which is consistent with previous results [31], also showing alpha diversity indexes increased at higher in vitro simulated pH (7.9–8.1) relative to lower pH (5.9–6.1). It has been suggested that microbial cell density and alpha diversity increase from the proximal to distal colon where the microbial fermentation towards the breakdown of protein sources result in increased pH when the carbohydrate sources are depleted [5], possibly explaining the higher alpha diversity with increased pH conditions found in the present study. The significant reduction in alpha diversity (observed species and Shannon index) of all four substrate groups relative to inoculum (Fig. 1A-B) might result from adding a single carbon source in the medium under simplified in vitro simulations, which contrasts with the human diet containing a range of complex components [32]. In our study, colonic pH significantly influenced the microbiota composition, but to a smaller degree relative to the effect of donor and substrate (Fig. 1D). It is well established that specific substrates could boost the population of specific bacteria but the microbial patterns also showed clear inter-individual variability [19]. Our findings showed that substrate has pronounced impact on bacterial community relative to the pH, which is inconsistent with a previous report [23], and the explanation might be found from the complexity of the individual bacterial composition and interactions with different diet components in vivo.

The high relative abundance of *Actinobacteria* members such as *Bifidobacterium adolescentis* and other bifidobacteria as well as *Collinsella aerofaciens* after fermentation when using inulin, lactose, GOS, or FOS (Fig. 2) as substrate confirmed the bifidogenic effects of these common prebiotics [27, 33]. For example, *Collinsella aerofaciens* is well-known for fermenting a variety of carbohydrates and producing SCFAs in the human colon [34]. Besides, we observed that the high abundance of *Bifidobacterium* spp. at low pH was associated with inulin and FOS substrates for donor 2 and donor 3 (Fig. 2 and Fig. S4), indicating that the proliferation of *Bifidobacterium* spp. was closely related to colonic pH. While inulin and FOS for at least two of the donors led to increased *Bifidobacterium* spp. abundance, this was not the case for lactose and GOS. It can be speculated that other microbiome members simply are more efficient in utilizing lactose; GOS on the other hand has in other studies been found to promote bifidobacteria [35], but we did not observe this effect – a possible explanation being that different bifidobacteria differ in their ability to utilize GOS efficiently with the donors in the present study harbouring bifidobacteria, that are not well-adapted for utilizing GOS [36]. This is in agreement with previous in vitro studies showing higher *Bifidobacterium* spp. relative abundance at pH 5.5 compared to pH 6.5 with arabinoxylan [19] and inulin [11] as a single carbohydrate source. *Bifidobacterium* spp. enzyme activity can differ in various carbohydrate fermentations with different pH conditions in vitro [12], and possibly a similar mechanism is in play in the present study. It can be speculated that the bifidogenic effect of substrates like inulin observed in vivo [37] might result from the synergistic effect of such substrates leading to increased SCFA production that lowers colonic pH, which again renders *Bifidobacterium* metabolism more efficient (and/or inhibits competitors occupying the same niche). This also suggests the importance of pH in influencing not only bacterial activity but also their abundance in the gut, as the enzyme activity, preference for specific substrates, and protein synthesis can be regulated in the face of even slight environmental differences [38].

Also the production of SCFA differed in a donor and substrate-dependent manner, but less pronounced than what was observed for the GM profiles (Figs. 2, 3, 4, and S6). Similarly, Reichardt et al. [19] also found that the GM profile was more donor-dependent than the SCFA profile. This is the result of a large number of colonic bacteria capable of utilizing substrates and producing acetate, propionate, and butyrate as three main fermentation products, thus leading to functional redundancy with respect to metabolite production relative to the GM composition. For inulin, GOS, and FOS the SCFA production increased with higher simulated colonic pH. This is in support of previous findings [19] that especially acetate and propionate production is higher at pH 6.5 relative to pH 5.5, possibly due to environmental pH influencing either gene expression and/or enzyme activity of the relevant pathways [39].

The increased butyrate production from inulin at the higher simulated colonic pH-levels positively correlated with the relative abundance of the butyrate-producers *Butyricimonas* (Fig. 5A) [40] and *Christensenella* (Fig. 3) [41, 42]. Consistent with previous findings, we observed positive correlations between butyrate production and the abundance of *Coprococcus* [43] and *Lachnospiraceae* [44] members, which have previously been reported as potentially butyrogenic bacteria. LaBouyer et al. [15] reported a negative relationship between pH and butyrate in human feces. This is seemingly in contrast with in vitro findings but likely reflects that in vivo fecal SCFA concentrations represent the balance between production, absorption (by the host), and cross-feeding to other microbes, i.e. of acetate to butyrate producers. Our result might suggest that butyrate production is more efficient at higher pH conditions, but the production of precursors like acetate and the end product itself, butyrate, reduces the pH in vivo. Propionate production and *Phascolarctobacterium*, *Bacteroides*, and *Rikenellaceae* relative abundance were linked and both enhanced at the higher colonic pH levels tested for the GOS and FOS groups. *Rikenellaceae* members are prominent fiber fermenters in the human colon resulting in the production of propionate [45]. *Phascolarctobacterium* spp. are succinate-metabolizing bacteria co-existing with *Bacteroides* members in the gut and producing substantial amounts of acetate and/or propionate which is in accordance with the strong association between these factors observed in Fig. 5B [17, 19, 46]. Of note, the precursors of SCFA production, lactate and formate, do not usually accumulate in the gut under in vivo conditions [47] but are utilized by gut microbes. Lactate accumulation in GOS-based fermentations at low in vitro simulated colonic pH matched with a corresponding reduction of propionate levels, which might be explained by the inter-species competition between lactate-producing *Bifidobacterium* [20, 22] and some other potential lactate–utilizing members [21].

Various in vitro models simulating the large intestine have been developed over the years. They span from simple batch fermentations inoculated with fecal matter but without pH-control to advanced models such as the SHIME and TIM-2 systems [48]. All models come with advantages and disadvantages. Simple batch models for instance offer very high throughput at low prices, but come with the caveat that due to lacking pH control acid stress (low pH) becomes a pronounced problem within hours. The more advanced models on the other hand have a very low throughput and the cost is high. Importantly absorption of SCFAs over the epithelium by the human host is generally not simulated in existing in vitro colon models (with the TIM-2 model partly as an exception). In the present study, we utilized a semi-dynamic in vitro colon model, the CoMiniGut [49, 50], where pH is controlled, but the density of solid matter for instance will be markedly lower than in vivo*.* Cross-feeding between microbes will still influence SCFA concentration dynamics, but due to the lacking absorption of SCFAs, concentrations might increase to levels beyond what is seen in the gut if the substrate concentration is too high. In the present study, we used inoculum representing donors with relatively low (6.4), medium (6.8), and high (7.2) fecal pH. Fecal samples were used as inoculum. Importantly, fecal samples do not perfectly represent the colonic microbiome composition and there will inevitable be strains and functions that will be overrepresented in one sample type over the other [51, 52], but for ethical and practical reasons (e.g. no invasive sampling) fecal samples are still the inoculum of choice for practically all in vitro simulated colonic fermentation setups [48]. The starting pH of each run was then adjusted to either a low (5.2), medium (5.6), or high (6.0) value (all donors tested at all pH-gradients). One can speculate, that a fecal microbiome originating from a high pH fecal sample will not have microbial composition optimally suited from e.g. a low starting pH – but in the present study, the main purpose was also merely to test how different pH-regimes influence different fecal inoculums.

## Conclusion

In summary, we found that the influence of colonic pH gradients on SCFA production is linked to concurrent changes in the bacterial community profile in a donor and substrate-dependent manner. Our results indicate that colonic substrates such as dietary fibres influence GM composition and metabolite production, not only by being selectively utilized by specific microbes, but also because of their SCFA production, which in turn also influences colonic pH and overall GM composition and activity.

## Materials and methods

### Materials

FOS (F8052, purity ≥ 90%) was purchased from Sigma-Aldrich Chemical Co. (St. Louis, MO, USA). Inulin (YI012742001, from chicory, DP: 2–60, purity ≥ 95%) and GOS (OG321341901, purity ≥ 95%) were purchased from Biosynth Carbosynth (Berkshire, UK). Lactose (VM922157008, purity ≥ 98%) was purchased from Merck KGaA (Darmstadt, Germany). All reagents used in phosphate-buffered saline (PBS) and basal colon media (BCM) [49] were of analytical grade and bought from Sigma-Aldrich or Merck.

### Fecal samples collection and pH determination

Healthy adult anonymous donors (18–60 years of age) donated fecal samples for the study. No dietary restrictions were imposed on the donors. None had received antibiotic treatments during the past three months before donating. Informed consent to participate was obtained from all donors, and the experiment was carried out with approval from the Ethical Committee of the Capital Region of Denmark (registration number H-20028549). The fecal samples were handled under anaerobic conditions in an anaerobic chamber (AALC model, Coy Laboratory Product, atmosphere ~ 93% (v/v) N_2_, 2% H_2_, 5% CO_2_). Individual stool samples were transferred to the chamber within minutes after collection and homogenized with sterilized PBS/20% glycerol (v/v) at a ratio of 1:1 for 120 s using the Stomacher (Stomacher 400; Seward, Worthing, UK). The glycerol stocks were aliquoted into cryo-vials and stored at -60 ℃ before use as fecal inoculum for the fermentations. One part of the individual feces was mixed with distilled water at a ratio of 1:9 after collection, and the pH of the mixture was determined with a pH meter calibrated on the same day (FC240B, Hanna Instruments, UK). Among these fecal samples, three donors with fecal pHs of 6.4, 6.8, and 7.2 representing low, medium and high fecal pH-levels typically found in humans [3–5] were selected for the study.

### In vitro colonic fermentations

**Fig. 6:**
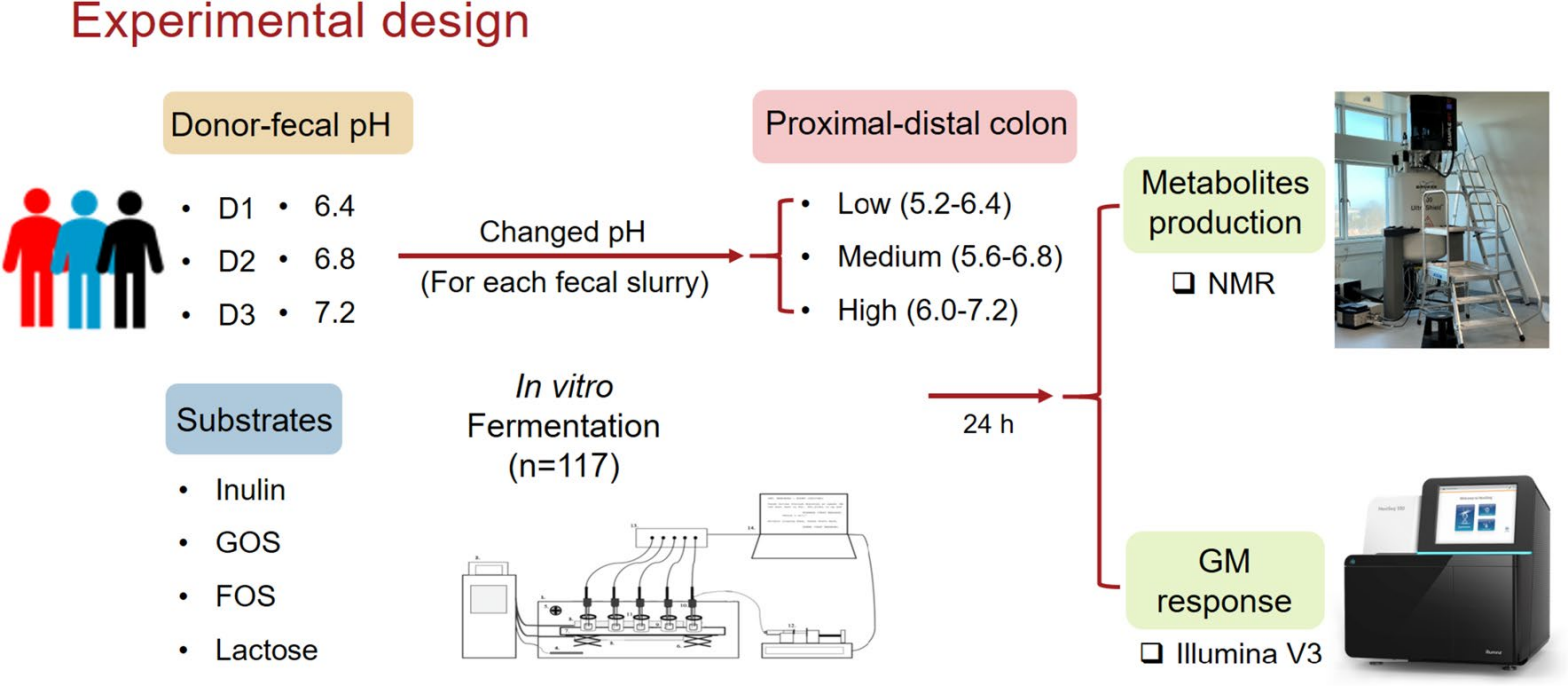
Diagram illustrating the experimental design. Fresh stool samples from three healthy donors with different fecal pH values (6.4, 6.8, and 7.2, respectively) were collected, and three dynamic pH programs during 24 h of fermentation were designed which represent the low (5.2-6.4), medium (5.6-6.8) and high (6.0-6.8) pH changes from the proximal to the distal colon. Single substrates (inulin, lactose, GOS, or FOS) were added to each chamber for colonic fermentation. Metabolite outcomes after 24 h of fermentation for each fecal slurry under the corresponding pH and abnormal colonic pHs were detected by ^1^H NMR, and GM shifts were traced using the V3 region of the 16S rRNA gene amplicon sequencing by Illumina, NextSeq. D, donor

The fermentations were performed using the previously described CoMiniGut in vitro colon model with minor modifications to the procedure [49, 50]. The experimental design is summarized in Fig. 6. All tested fermentation conditions were conducted in triplicates. Briefly, fecal glycerol stocks from three donors were thawed and further diluted with sterilized PBS in a ratio of 1:4. All fermentations were carried out in 4.5 mL BCM (2 g/L yeast extract, 2 g/L peptone, 0.5 g/L bile salts, 0.1 g/L NaCl, 0.04 g/L K_2_HPO_4_, 0.04 g/L KH_2_PO_4_, 0.01 g/L CaCl_2_∙6H_2_O, 0.01 g/L MgSO_4_∙7H_2_O, 2 g/L NaHCO_3_, 0.5 g/L L-Cysteine HCl, 0.002 g/L hemin, 0.2% (v/v) Tween 80, and 0.001% (v/v) vitamin K1) containing 50 mg substrate (inulin, FOS, GOS, or lactose) and 0.5 mL fecal slurry. Anaerobic conditions during fermentation were achieved by positioning an anaerogen compact sachet (AN0010W; ThermoScientific, Waltham, MA, USA) in the reaction chamber. Resazurin-soaked indicators (Anaerobe Indicator Test; Sigma-Aldrich, St. Louis, MO, USA) were used to signal anaerobiosis. The starting pH of each fermentation was adjusted by adding 0.5 M of NaOH or 0.1 M of HCl until the desired pH was reached [49]. Three dynamic pH programs (low, 5.2 increasing to 6.4; medium, 5.6 increasing to 6.8 and high, 6.0 increasing to 7.2) simulating the pH from the proximal colon to the distal colon for each fecal slurry were controlled by connecting the pH meter with a laptop running in Matlab scripts which regulates a multichannel syringe pump charged with syringes adjusting pH by adding 2.5 µL aliquots of 1 mM of NaOH pr. bolus. After 24 h of fermentation, fermentates with individual inoculum under different pH programs were collected for further metabolite determination and GM analysis. Samples were stored at -60 °C until further analysis.

### ^1^H NMR spectroscopic analysis and metabolite quantification

^1^H NMR spectroscopy of collected fermentates was performed using Bruker Avance IVDr NMR spectrometer (Bruker BioSpin, Rheinstetten, Germany) equipped with a 5 mm ^1^H-optimized double resonance broad-band probe and operating at a frequency of 600.13 MHz for ^1^H. Samples for ^1^H NMR spectroscopy were prepared according to a procedure described by He et al. [53] with minor modifications. In brief, 500 µL of fermentation sample was vortexed and filtered by centrifugation at 4 ℃, 14 000 g for 30 min using Amicon Ultrafilters (Merck Millipore Ltd., Cork, Ireland), and 300 µL supernatant was mixed with 300 µL phosphate buffer in D_2_O (pH 7.4) containing trimethylsilylpropanoic acid (TSP) (0.01% w/w) in 5 mm NMR tubes. ^1^H NMR spectra were obtained at a temperature of 300 K using a one-dimensional NOESY pulse sequence with following acquisition parameters: relaxation delay: 4 s, spectral width: 7212 Hz, data points: 32 k, and number of scans: 32. The free induction decays with a line-broadening factor (0.3 Hz) were adopted before Fourier transformation. Phase adjustments and baseline correction of obtained ^1^H NMR spectra were performed in the Topspin 3.6.2 software. Multivariate data analysis (MVDA) including PCA and supervised orthogonal projections to latent structures discriminant analyses (OPLS-DA) were conducted on binned ^1^H NMR spectra (bin width = 0.005 ppm) after removal of the residual water signal (4.75–7.90 ppm). The S-line plots of the OPLS-DA models were employed to examine the spectral differences between the low and high pH programs of the total samples and fermentates with inulin as a carbohydrate source, respectively. In addition, identification and quantification of metabolites from the ^1^H NMR spectra obtained for the 24 h fermentates were employed by Chenomx (Version 8.6, Chenomx Inc., Alberta, Canada). For metabolite production including the total SCFA, a t-test was applied for pairwise comparison of different pH programs in each substrate after 24 h of fermentation.

### DNA extraction, library preparation, and Illumina sequencing

The microbiome composition of in vitro colonic fermentations as well as the fecal inoculums were characterized by 16S rRNA gene amplicon sequencing. DNA extraction was performed using the Micro Bead beat AX kit (A&A Biotechnology, Poland) following the manufacturer’s instructions. Qubit dsDNA BR Assay Kit (Thermo Fisher Scientific Inc., Waltham, USA) and NanoDrop ND-1000 Spectrophotometer (NanoDrop Technologies Inc., Wilmington, USA) were used for determining the concentration and purity of the extracted DNA. The V3 hypervariable region of the 16S rRNA gene was amplified using primers compatible with the Nextera Index Kit (Illumina, San Diego, CA, USA) NXt_338_F:5'- TCGTCGGCAGCGTCAGATGTGTATAAGAGACAGACWCCTACGGGWGG CAGCAG-3' and NXt_518_R: 5'-GTCTCGTGGGCTCGGAGATGTGTATAAGAGACAGATTACCGCG GCTGCTGG-3'. The PCR1 reaction mix is composed of 5 µl of PCRBIO buffer and 0.25 µl of PCRBIO HiFi polymerase (PCR Biosystems Ltd., London, United Kingdom), 0.5 µl of primer mix, 1 µl of BSA and formamide, nuclease-free water and 5 µl of genomic DNA (5 ng/ µl) to a total volume of 25 µl. The PCR thermal conditions were set as follows: the denaturation started at 95 °C for 5 min and was followed by 33 cycles of 95 °C for 15s, 55 °C for 20s, and 72 °C for 20s. Finally, the temperature was maintained at 72 °C for 4 min for elongation. PCR1 products were verified by agarose gel electrophoresis before barcoding in PCR2. The PCR2 mix contained 5 µl of PCRBIO buffer, 0.25 µl of polymerase, 4 µl of barcode primer, nuclease-free water, and PCR1 product (2 µl) to a total volume of 25 µl. The PCR2 thermal conditions were as follows: denaturation at 95 °C for 1 min; 13 cycles of 95°C for 15s, 55°C for 15s, 72°C for 15s; final elongation at 72 °C for 5 min [54]. The PCR2 products were purified using AMPure XP beads (Beckman Coulter Genomic, CA, USA) and pooled in equimolar concentrations for sequencing using an Illumina NxtSeq platform (2 × 150 bp chemistry).

### Bioinformatics and statistics

The raw Illumina data set containing pair-ended reads with matching quality scores were merged and trimmed in the USEARCH pipeline [54] using fastq_mergepairs and fastq_filter scripts. UNOISE3 [55] was used to build zOTUs, and the Greengenes (13.8) 16S rRNA gene collection [56] was used as a reference database for taxonomy assignment. Statistical analysis and plot visualization were performed by R (v 4.1.3). The feature table, taxonomy, metadata, and tree file were imported through phyloseq in R [57]. For diversity analysis, samples were rarefied to 100,000 reads with the function “rarefy_even_depth” in phyloseq. A t-test was conducted to determine the statistical differences in alpha diversity (observed species and Shannon index) between different pH programs of the specific substrates, and PERMANOVA was employed for determining GM structural changes based on Bray–Curtis dissimilarity and Jaccard similarity matrixes, respectively. Distance-based redundancy analysis (db-RDA) based on Bray–Curtis metrics was performed to discern the variance explained by colonic pH, and the effect size of other factors such as donor, substrate, and pH was tested by the function “adonis”. For GM composition, taxa were agglomerated at the species level, and the collapsed features at the species level as well as the summarized abundance of *Bifidobacterium* among pH conditions for each substrate were visualized with bar plots in “ggplot2” [58]. Differential abundant taxa between different pH programs were found by Deseq2 [59] with an adjusted *p*-value lower than 0.05, and differences in the abundance of the top 20 taxa with the lowest adjusted *p*-values were plotted in a heatmap using the R package “complexheatmap” [60]. For microbial co-occurrence analysis, species with a mean relative abundance of more than 1.5% were chosen for analysis, and the chord diagram was visualized by R package “circlize” with Spearman's rank correlation coefficient of more than 0.7. Pearson correlation analysis between metabolite concentrations and the relative abundance of microbial community members was performed with the package “Rhea” [61] and R package “corrplot” was used to visualize the correlation coefficients. The minimal prevalence (among the sequenced samples) of one given microbial taxa was set to 30%.

### Supplementary Information

**Supplementary Material 1.**

**Supplementary Material 2.**

## Data Availability

The raw sequences data produced in this study is released through the NCBI Sequence Read Archive under BioProject accession number PRJNA932979. Analytic codes are available upon request.

## Abbreviations

BCM: Basal Colon Media
Db-RDA: Distance-based Redundancy Analysis
Deseq2: Differential abundance analysis
DP: Degree of polymerization
FOS: Fructooligosaccharides
GM: Gut Microbiota
GOS: Galacto-oligosaccharides
MVDA: Multivariate Data Analysis
OPLS-DA: Orthogonal Projections to Latent Structures Discriminant Analysis
PBS: Phosphate-buffered Saline
SCFA: Short-chain Fatty Acids
TSP: Trimethylsilylpropanoic acid
zOTUS: Zero radius Operational Taxonomic Units
