# Supplementary material for: The effect of in vitro simulated colonic pH gradients on microbial activity and metabolite production using common prebiotics as substrates
Source: BMC Microbiol. 2024 Mar 11;24:83. doi: 10.1186/s12866-024-03235-2 (PMC10926653; doi:10.1186/s12866-024-03235-2)
Supplement: Supplementary file 1 — Supplementary Material 1. [file 12866_2024_3235_MOESM1_ESM.docx]

**Supplementary file**

Table S1. The Q^2^ of OPLS-DA models constructed to discriminate the gut metabolome from different pH groups.

| Comparison | Q^2^ |
| --- | --- |
| Low vs Medium | 0.28 |
| Low vs High | 0.82 |
| Medium vs High | 0.21 |

Q^2^ > 0.5 indicates that the OPLS-DA model is robust.


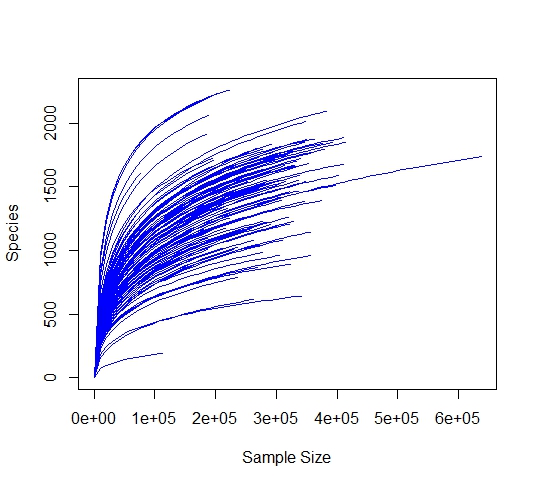


**Fig.S1** Rarefaction curves for the microbiome data in this study.

**
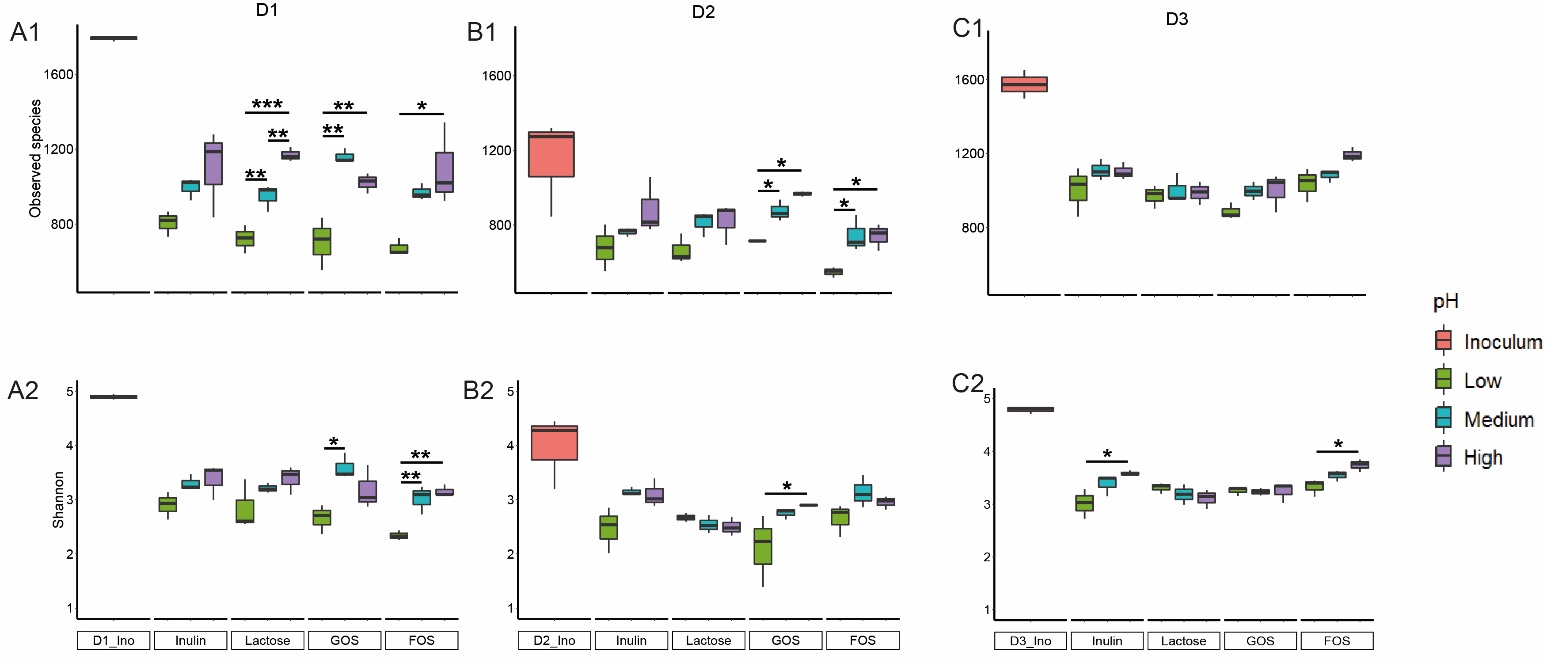
**

**Fig. S2** The influence of colonic pH on individual GM alpha diversity after 24 h of fermentation. A-C represents D1, D2, and D3, and 1-2 represents Observed species and Shannon index. A t-test was applied to determine the influence of pH on alpha diversity for each inoculum with a single substrate. Benjamin-Hochberg FDR (false discovery rate) correction was adopted for multiple testing. Significant differences between changed colonic pH are labelled with * (*p* < 0.05), ** (*p* < 0.01) and *** (*p* < 0.001), respectively. D, donor.

**
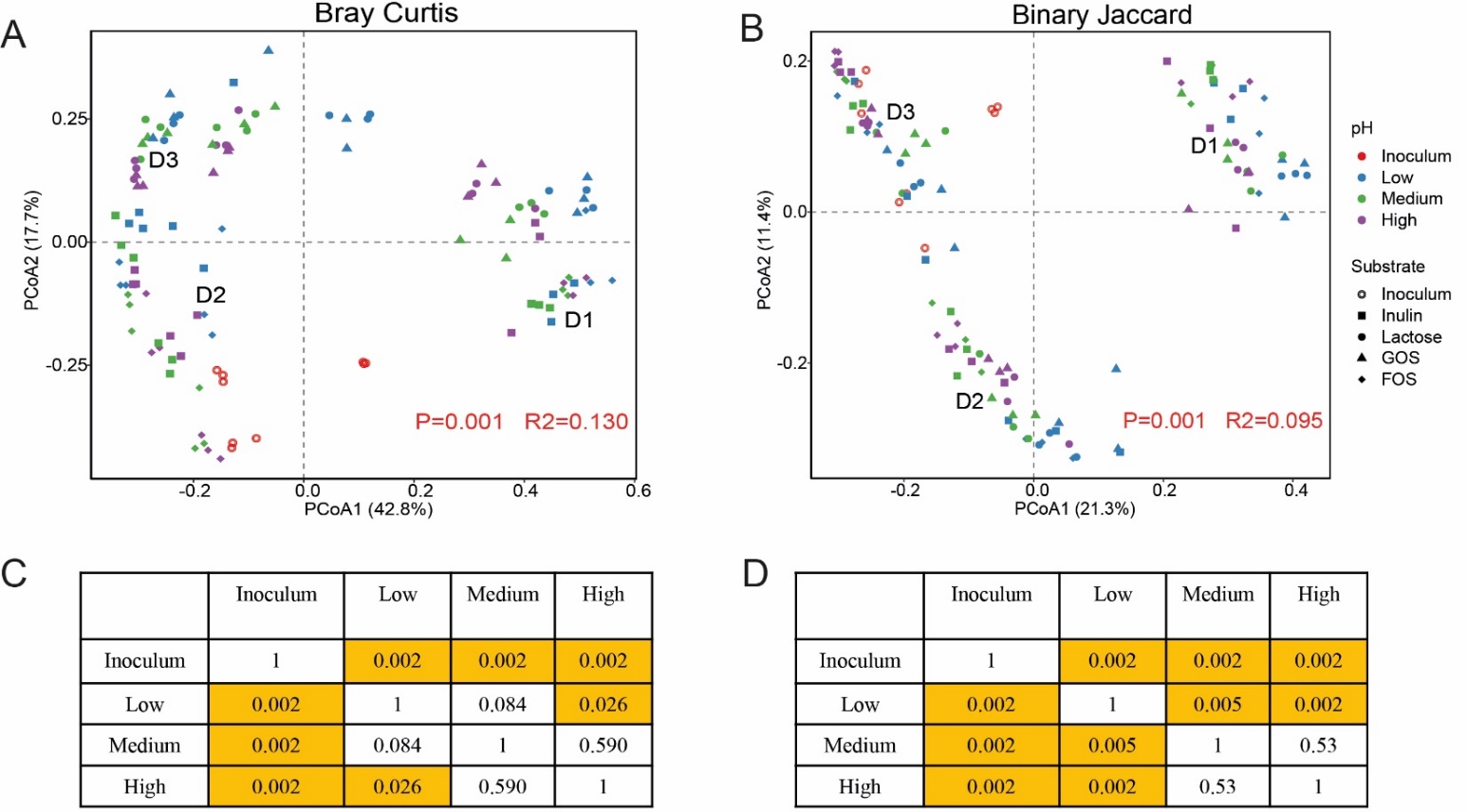
**

**Fig. S3** PCoA and pairwise PERMANOVA tests on Bray Curtis (A and C) and Binary Jaccard metrics (B and D).


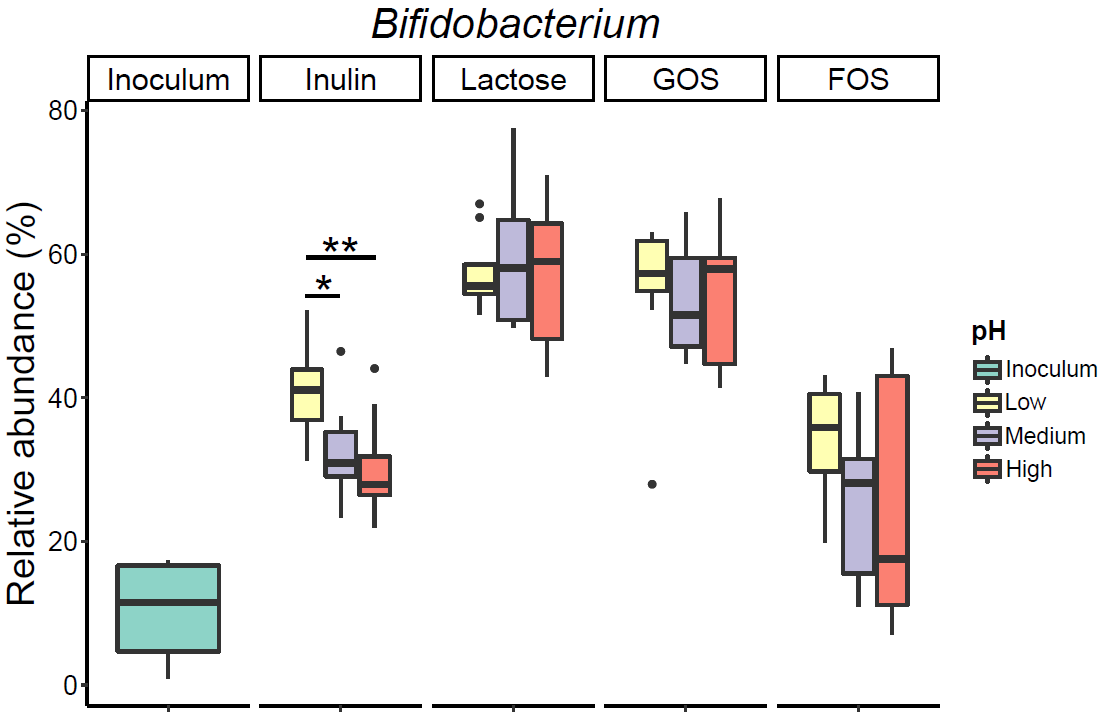


**Fig. S4** The influence of colonic pH on *Bifidobacterium* (relative abundance) after 24 h of fermentation using different substrates. A t-test was applied to determine the influence of pH on *Bifidobacterium* with a single substrate. Benjamin-Hochberg FDR (false discovery rate) correction was adopted for multiple testing. Significant differences between changed colonic pH are labelled with * (*p* < 0.05), ** (*p* < 0.01) and *** (*p* < 0.001), respectively.


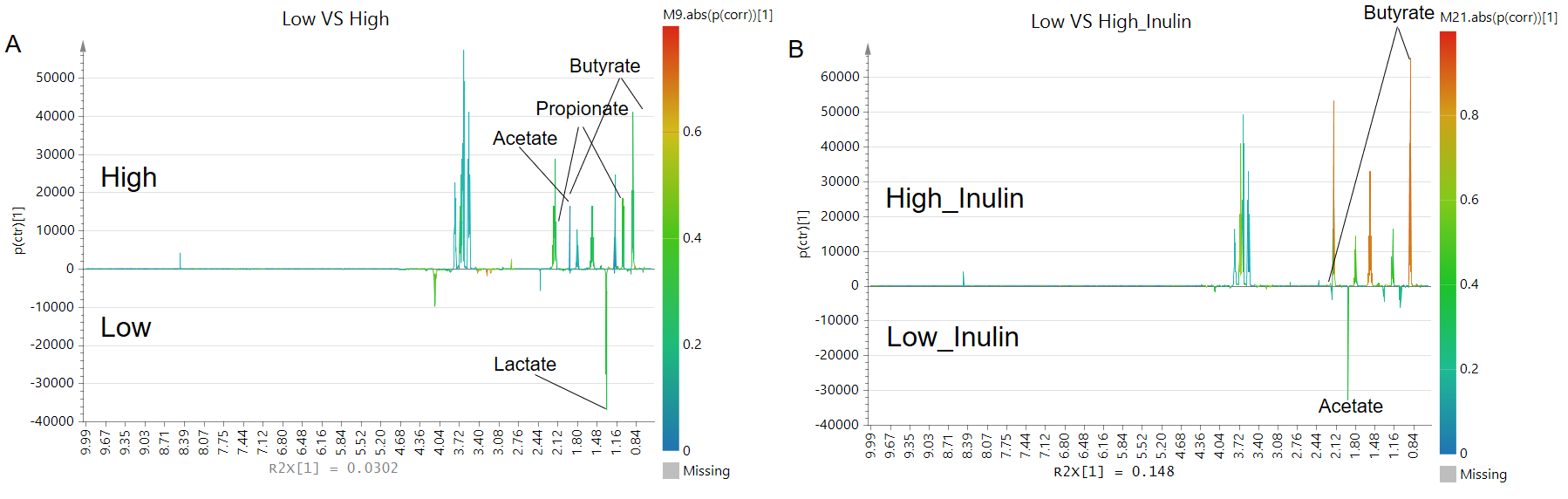


**Fig. S5** S-line plot of OPLS-DA visualizing the differences in the NMR metabolite profiles of low and high colonic pH (A, Q^2^ = 0.82) and only inulin (B, Q^2^ = 0.55).


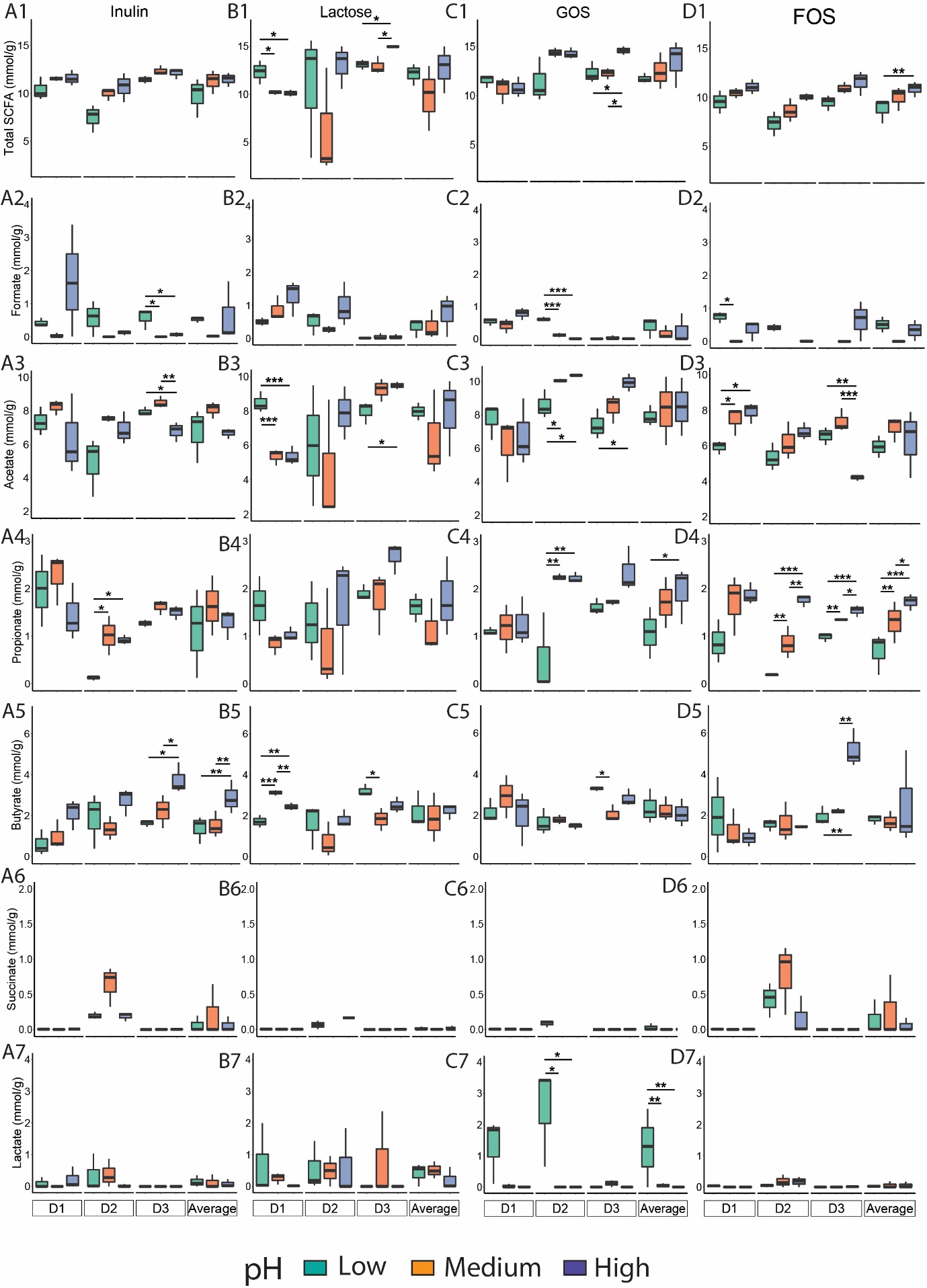


**Fig. S6** The influence of colonic pH on individual metabolite production (in mmol per gram of substrate) after 24 h of fermentation. A-D represents substrate inulin, lactose, GOS, and FOS, respectively, and 1-7 represents total SCFA, formate, acetate, propionate, butyrate, succinate, and lactate production, respectively.. A t-test was applied to determine the influence of pH on individual metabolite production with a single substrate. Benjamin-Hochberg FDR (false discovery rate) correction was adopted for multiple testing. Significant differences between changed colonic pH are labelled with * (*p* < 0.05), ** (*p* < 0.01) and *** (*p* < 0.001), respectively. D, donor.
